# Supplementary material for: Fine-scale movement patterns and habitat selection of little owls (Athene noctua) from two declining populations
Source: PLoS One. 2021 Sep 27;16(9):e0256608. doi: 10.1371/journal.pone.0256608 (PMC8476024; doi:10.1371/journal.pone.0256608)
Supplement: S1 Fig — Examples of simulated correlated random walks (grey lines) generated based upon real little owl trajectories (red lines). The nest location is shown in each case as a yellow dot. We generated 10× the number of available data points (via random walks) than we had GPS positions (although here we only show the same number of used and available data for reasons of clarity and comparability). (DOCX) [file pone.0256608.s001.docx]

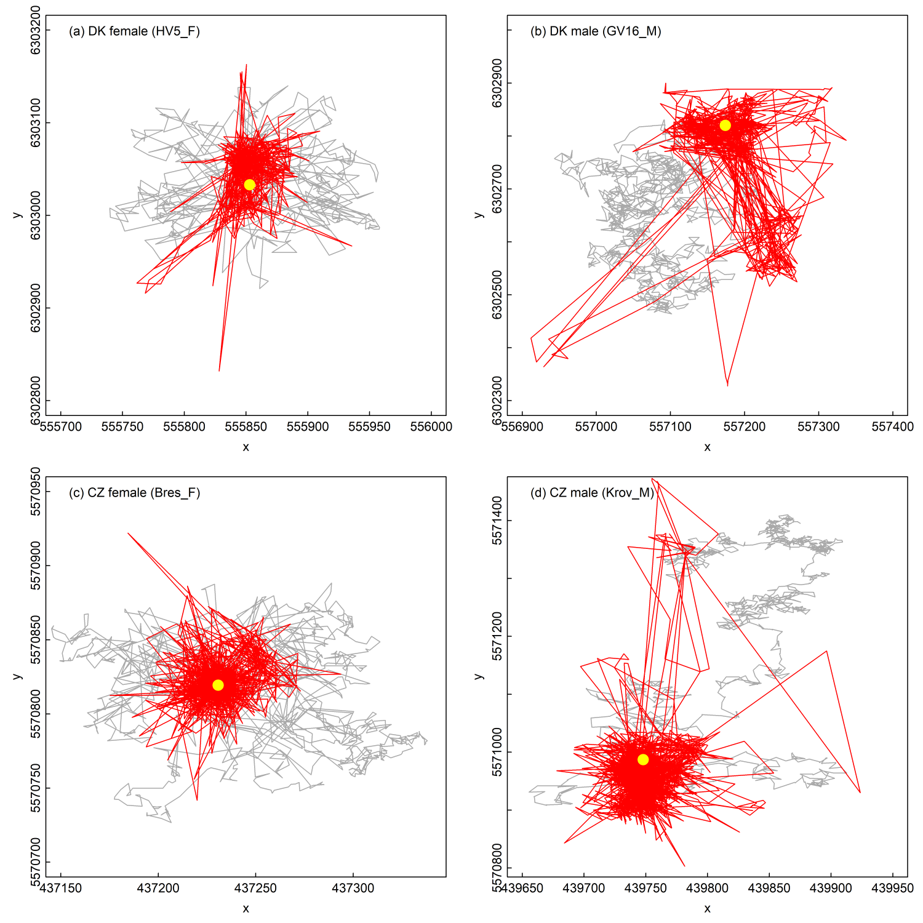


**S1 Fig. Examples of simulated correlated random walks (grey lines) generated based upon real little owl trajectories (red lines).** The nest location is shown in each case as a yellow dot. We generated 10× the number of available data points (via random walks) than we had GPS positions (although here we only show the same number of used and available data for reasons of clarity and comparability).
