# Supplementary material for: Fine-scale movement patterns and habitat selection of little owls (Athene noctua) from two declining populations
Source: PLoS One. 2021 Sep 27;16(9):e0256608. doi: 10.1371/journal.pone.0256608 (PMC8476024; doi:10.1371/journal.pone.0256608)
Supplement: S2 Fig — The effect size of the interaction between the land cover type and distance from the nest on the relative probability of use, shown separately for male little owls from (a) Denmark and (b) the Czech Republic. Shading indicates 95% confidence intervals. (DOCX) [file pone.0256608.s002.docx]

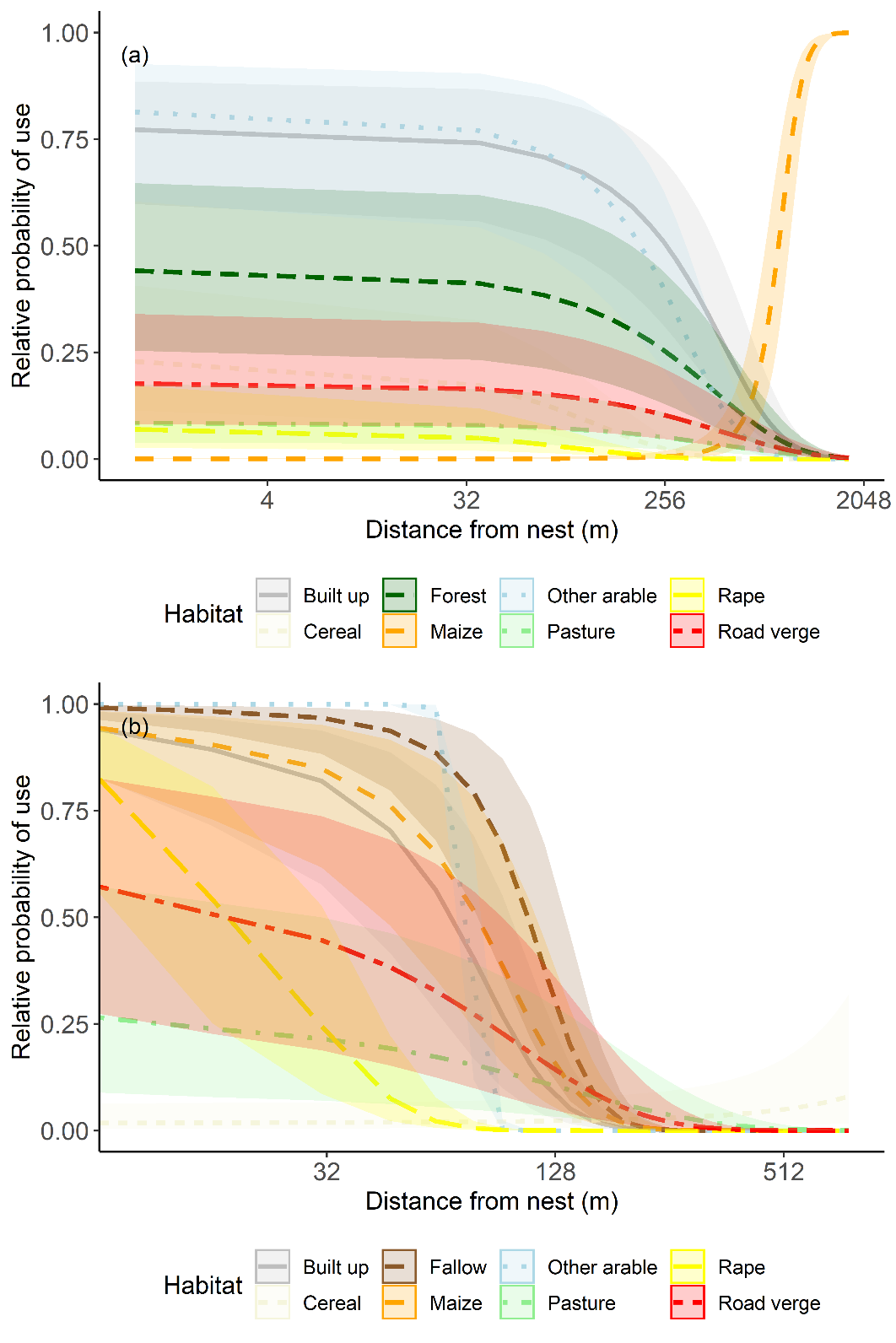


**S2 Fig.** The effect size of the interaction between the land cover type and distance from the nest on the relative probability of use, shown separately for male little owls from (a) Denmark and (b) the Czech Republic. Shading indicates 95% confidence intervals.
