# Supplementary material for: Fine-scale movement patterns and habitat selection of little owls (Athene noctua) from two declining populations
Source: PLoS One. 2021 Sep 27;16(9):e0256608. doi: 10.1371/journal.pone.0256608 (PMC8476024; doi:10.1371/journal.pone.0256608)
Supplement: S1 Table — R2m = marginal R2 (fixed effects only), R2c = conditional R2 (fixed effects and random intercept). (DOCX) [file pone.0256608.s003.docx]

**S1 Table. Overview of the best, full and intercept only model for the analyses of little owl distance from the nest and displacement per hour (for all data and separately for Danish and Czech owls).** R^2^m = marginal R^2^ (fixed effects only), R^2^c = conditional R^2^ (fixed effects and random intercept).

| Analysis/Dependent variable | Fixed effects | df | logLik | AICc | delta AIC | AIC weight | R^2^m | R^2^c |
| --- | --- | --- | --- | --- | --- | --- | --- | --- |
| Distance from the nest (all data) | |  |  |  |  |  |  |  |
| Best | Area + Sex + Time of night + Temperature + Sex × Time of night | 22 | -20172 | 40388 | 0 | 0.998 | 0.27 | 0.33 |
| Full | Area + Number of chicks + Precipitation + Temperature + Sex + Time of night + Sex x Time of night | 25 | -20175 | 40401 | 12 | 0.002 | 0.27 | 0.34 |
| Null | Intercept only | 3 | -21072 | 42151 | 1763 | 0.000 |  | 0.26 |
|  |  |  |  |  |  |  |  |  |
| Distance from the nest (Denmark) | |  |  |  |  |  |  |  |
| Best | Sex + Time of night + Sex × Time of night | 20 | -9965 | 19971 | 0 | 0.920 | 0.31 | 0.41 |
| Full | Number of chicks + Sex + Time of night + Precipitation + Sex × Time of night | 23 | -9965 | 19976 | 5 | 0.080 | 0.30 | 0.44 |
| Null | Intercept only | 3 | -11214 | 22434 | 2464 | 0.000 |  | 0.12 |
|  |  |  |  |  |  |  |  |  |
| Distance from the nest (Czech Republic) | |  |  |  |  |  |  |  |
| Best | Precipitation + Temperature + Sex + Time of night + Sex x Time of night | 22 | -9062 | 18169 | 0 | 0.880 | 0.19 | 0.24 |
| Full | Number of chicks + Precipitation + Temperature + Sex + Time of night + Sex x Time of night | 23 | -9063 | 18173 | 4 | 0.120 | 0.20 | 0.24 |
| Null | Intercept only | 3 | -9444 | 18893 | 724 | 0.000 |  | 0.22 |
|  |  |  |  |  |  |  |  |  |
| Number of daily foraging trips (>200 m from nest) | |  |  |  |  |  |  |  |
| Best | Area + Sex | 4 | -71 | 150 | 0 | 0.757 | 0.43 | 0.53 |
| Full | Area + Sex + Area x Sex | 5 | -71 | 153 | 3 | 0.213 | 0.43 | 0.53 |
| Null | Intercept only | 2 | -76 | 157 | 6 | 0.031 |  | 0.46 |
|  |  |  |  |  |  |  |  |  |
| Foraging trip (>200 m from nest) duration | |  |  |  |  |  |  |  |
| Best/Full | Area + Sex + Area x Sex | 6 | -425.7 | 864.3 | 0 | 1.000 | 0.3 | 0.43 |
| Null | Intercept only | 3 | -440.9 | 888.1 | 24 | 0.000 |  | 0.40 |
|  |  |  |  |  |  |  |  |  |
| Hourly displacement (all data) | |  |  |  |  |  |  |  |
| Best/Full | Number of GPS positions + Area + Number of chicks + Precipitation + Temperature + Sex + Time of night + Sex x Time of night | 25 | -2290 | 4634 | 0 | 1.000 | 0.68 | 0.70 |
| Null | Intercept only | 3 | -2551 | 5108 | 473 | 0.000 |  | 0.36 |
|  |  |  |  |  |  |  |  |  |
| Hourly displacement (Denmark) | |  |  |  |  |  |  |  |
| Best/Full | Number of GPS positions + Number of chicks + Precipitation + Temperature + Sex + Time of night + Sex x Time of night | 24 | -862 | 1782 | 0 | 1.000 | 0.65 | 0.65 |
| Null | Intercept only | 3 | -1060 | 2126 | 343 | 0.000 |  | 0.20 |
|  |  |  |  |  |  |  |  |  |
| Hourly displacement (Czech Republic) | |  |  |  |  |  |  |  |
| Best/Full | Number of GPS positions + Number of chicks + Precipitation + Temperature + Sex + Time of night + Sex x Time of night | 24 | -1230 | 2515 | 0 | 1.000 | 0.74 | 0.78 |
| Null | Intercept only | 3 | -1476 | 2959 | 444 | 0.000 |  | 0.14 |
