# Supplementary material for: Fine-scale movement patterns and habitat selection of little owls (Athene noctua) from two declining populations
Source: PLoS One. 2021 Sep 27;16(9):e0256608. doi: 10.1371/journal.pone.0256608 (PMC8476024; doi:10.1371/journal.pone.0256608)
Supplement: S2 Table — Results are shown for the analyses of all data and separately for Danish (GPS-tagged in 2019) and Czech little owls (GPS-tagged in 2020). Informative values are given in bold (i.e., 95% confidence intervals not overlapping zero). (DOCX) [file pone.0256608.s004.docx]

**S2 Table. Effect size (β), standard error (SE), lower 95% confidence interval (LCI) and upper 95% confidence interval (UCI) of explanatory variables for the analyses of the (1) distance from nest and (2) displacement per hour.** Results are shown for the analyses of all data and separately for Danish (GPS-tagged in 2019) and Czech little owls (GPS-tagged in 2020). Informative values are given in bold (i.e., 95% confidence intervals not overlapping zero).

| Variable | β | SE | LCI | UCI |  | β | SE | LCI | UCI |  | β | SE | LCI | UCI |
| --- | --- | --- | --- | --- | --- | --- | --- | --- | --- | --- | --- | --- | --- | --- |
|  | All data | | | |  | Denmark | | | |  | Czech Republic | | | |
| (1) Distance from nest | |  |  |  |  |  |  |  |  |  |  |  |  |  |
| Intercept | **2.09** | **0.20** | **1.69** | **2.49** |  | **3.39** | **0.25** | **2.90** | **3.87** |  | **2.55** | **0.3** | **1.97** | **3.13** |
| Area Denmark | **1.00** | **0.20** | **0.62** | **1.39** |  |  |  |  |  |  |  |  |  |  |
| Time 2200 | **0.90** | **0.07** | **0.76** | **1.04** |  | **0.45** | **0.08** | **0.29** | **0.60** |  | 0.48 | 0.25 | 0 | 0.96 |
| Time 2300 | **1.66** | **0.07** | **1.52** | **1.80** |  | **2.38** | **0.08** | **2.22** | **2.53** |  | 0.05 | 0.25 | -0.43 | 0.54 |
| Time 0000 | **0.87** | **0.07** | **0.73** | **1.01** |  | **0.76** | **0.08** | **0.60** | **0.92** |  | 0.02 | 0.25 | -0.46 | 0.5 |
| Time 0100 | **0.77** | **0.07** | **0.62** | **0.91** |  | **0.68** | **0.08** | **0.53** | **0.84** |  | -0.14 | 0.25 | -0.62 | 0.35 |
| Time 0200 | **1.12** | **0.07** | **0.97** | **1.26** |  | **1.33** | **0.08** | **1.18** | **1.48** |  | -0.06 | 0.25 | -0.54 | 0.43 |
| Time 0300 | **1.65** | **0.07** | **1.50** | **1.79** |  | **2.04** | **0.08** | **1.88** | **2.19** |  | 0.33 | 0.25 | -0.15 | 0.82 |
| Time 0400 | **0.80** | **0.08** | **0.66** | **0.95** |  | 0.12 | 0.08 | -0.04 | 0.27 |  | **0.85** | **0.25** | **0.36** | **1.34** |
| Time 0500 | **-0.19** | **0.09** | **-0.37** | **-0.02** |  | **-0.28** | **0.09** | **-0.46** | **-0.10** |  | -0.2 | 0.36 | -0.9 | 0.51 |
| Precipitation yes |  |  |  |  |  |  |  |  |  |  | **0.16** | **0.03** | **0.1** | **0.22** |
| Temperature | **0.02** | **0.00** | **0.01** | **0.03** |  |  |  |  |  |  | **0.04** | **0.01** | **0.03** | **0.05** |
| Sex F | **0.61** | **0.28** | **0.06** | **1.16** |  | 0.67 | 0.48 | -0.27 | 1.60 |  | -0.72 | 0.71 | -2.12 | 0.67 |
| Time 2200 × Sex F | **-0.65** | **0.21** | **-1.06** | **-0.23** |  | -0.51 | 0.26 | -1.02 | 0.00 |  | 0.24 | 0.69 | -1.11 | 1.6 |
| Time 2300 × Sex F | **-1.54** | **0.21** | **-1.95** | **-1.12** |  | **-2.56** | **0.26** | **-3.07** | **-2.04** |  | 0.56 | 0.69 | -0.8 | 1.92 |
| Time 0000 × Sex F | **-1.58** | **0.21** | **-2.00** | **-1.16** |  | **-1.64** | **0.33** | **-2.28** | **-1.00** |  | -0.27 | 0.69 | -1.63 | 1.09 |
| Time 0100 × Sex F | **-1.44** | **0.21** | **-1.86** | **-1.02** |  | **-1.52** | **0.37** | **-2.24** | **-0.79** |  | -0.06 | 0.69 | -1.42 | 1.29 |
| Time 0200 × Sex F | **-1.61** | **0.21** | **-2.02** | **-1.19** |  | **-1.77** | **0.24** | **-2.24** | **-1.29** |  | -0.04 | 0.69 | -1.39 | 1.32 |
| Time 0300 × Sex F | **-1.88** | **0.21** | **-2.28** | **-1.47** |  | **-2.36** | **0.24** | **-2.82** | **-1.90** |  | -0.08 | 0.69 | -1.44 | 1.27 |
| Time 0400 × Sex F | **-1.01** | **0.21** | **-1.43** | **-0.59** |  | **-0.51** | **0.24** | **-0.98** | **-0.04** |  | -0.46 | 0.69 | -1.83 | 0.9 |
| Time 0500 × Sex F | 0.07 | 0.24 | -0.40 | 0.54 |  | -0.04 | 0.25 | -0.53 | 0.45 |  | 1.25 | 0.98 | -0.67 | 3.17 |
|  |  |  |  |  |  |  |  |  |  |  |  |  |  |  |
| (2) Hourly displacement | |  |  |  |  |  |  |  |  |  |  |  |  |  |
| Intercept | **-982** | **326** | **-1621** | **-342** |  | **-1452** | **387** | **-2210** | **-694** |  | **-851** | **359** | **-1555** | **-148** |
| Area Denmark | **554** | **102** | **355** | **753** |  |  |  |  |  |  |  |  |  |  |
| Number of chicks | **175** | **71** | **36** | **314** |  | **281** | **98** | **89** | **474** |  | 121 | 86 | -48 | 291 |
| Time 2200 | **316** | **141** | **40** | **592** |  | 214 | 224 | -225 | 653 |  | **377** | **133** | **116** | **638** |
| Time 2300 | **809** | **147** | **522** | **1097** |  | **1463** | **229** | **1014** | **1911** |  | 100 | 141 | -176 | 377 |
| Time 0000 | **417** | **148** | **126** | **707** |  | **709** | **231** | **257** | **1161** |  | 44 | 141 | -233 | 320 |
| Time 0100 | 271 | 148 | -18 | 560 |  | 443 | 231 | -10 | 896 |  | 10 | 135 | -256 | 275 |
| Time 0200 | **453** | **155** | **149** | **757** |  | **663** | **232** | **210** | **1117** |  | 105 | 146 | -182 | 392 |
| Time 0300 | **859** | **158** | **549** | **1168** |  | **1205** | **230** | **755** | **1655** |  | **396** | **151** | **100** | **692** |
| Time 0400 | **371** | **155** | **67** | **675** |  | 120 | 234 | -337 | 578 |  | **525** | **143** | **244** | **805** |
| Time 0500 | 91 | 162 | -226 | 407 |  | 83 | 272 | -451 | 617 |  | 89 | 143 | -192 | 370 |
| Hourly GPS positions | **20** | **2** | **16** | **24** |  | **32** | **5** | **22** | **42** |  | **23** | **2** | **20** | **26** |
| Precipitation | 0 | 80 | -156 | 157 |  | 303 | 244 | -174 | 781 |  | 75 | 64 | -51 | 200 |
| Temperature | 8 | 12 | -14 | 31 |  |  |  |  |  |  | 18 | 10 | -2 | 37 |
| Sex F | -48 | 295 | -627 | 530 |  | 6 | 530 | -1032 | 1045 |  | -125 | 269 | -653 | 402 |
| Time 2200 × Sex F | -207 | 343 | -880 | 466 |  | -227 | 732 | -1663 | 1208 |  | -427 | 281 | -979 | 125 |
| Time 2300 × Sex F | -657 | 339 | -1321 | 7 |  | -1323 | 732 | -2758 | 111 |  | -98 | 279 | -645 | 449 |
| Time 0000 × Sex F | -500 | 335 | -1156 | 156 |  | -388 | 653 | -1668 | 891 |  | -274 | 277 | -818 | 270 |
| Time 0100 × Sex F | -315 | 340 | -981 | 351 |  | -326 | 739 | -1775 | 1122 |  | -207 | 277 | -751 | 337 |
| Time 0200 × Sex F | -445 | 332 | -1095 | 206 |  | -292 | 616 | -1500 | 916 |  | -239 | 280 | -788 | 309 |
| Time 0300 × Sex F | **-742** | **337** | **-1404** | **-81** |  | -1009 | 644 | -2271 | 252 |  | -473 | 281 | -1024 | 78 |
| Time 0400 × Sex F | -84 | 344 | -759 | 591 |  | 22 | 650 | -1252 | 1296 |  | -294 | 289 | -860 | 271 |
| Time 0500 × Sex F | 0 | 379 | -743 | 743 |  | -103 | 659 | -1395 | 1188 |  | 25 | 335 | -631 | 681 |
