# Supplementary material for: Fine-scale movement patterns and habitat selection of little owls (Athene noctua) from two declining populations
Source: PLoS One. 2021 Sep 27;16(9):e0256608. doi: 10.1371/journal.pone.0256608 (PMC8476024; doi:10.1371/journal.pone.0256608)
Supplement: S3 Table — Informative values are given in bold (i.e., 95% confidence intervals not overlapping zero). (DOCX) [file pone.0256608.s005.docx]

**S3 Table. Effect size (β), standard error (SE), lower 95% confidence interval (LCI) and upper 95% confidence interval (UCI) of explanatory variables for the analyses of the (1) number of daily little owl foraging trips >200 m from the nest and (2) duration of foraging trips >200 m from the nest. Informative values are given in bold (i.e., 95% confidence intervals not overlapping zero).**

| Variable | β | SE | LCI | UCI |
| --- | --- | --- | --- | --- |
| (1) Number of daily foraging trips (>200 m from the nest) | | | |  |
| **Intercept** | **-0.90** | **0.47** | **-1.95** | **-0.04** |
| Area Denmark | 0.58 | 0.33 | -0.13 | 1.33 |
| Sex Male | 1.40 | 0.48 | 0.49 | 2.44 |
|  |  |  |  |  |
| (1) Foraging trip duration |  |  |  |  |
| **Intercept** | **33.99** | **18.42** | **0.63** | **68.48** |
| Area Denmark | -18.94 | 26.06 | -67.73 | 28.23 |
| Sex Male | -17.75 | 20.94 | -55.97 | 19.99 |
| **Area Denmark x Sex Male** | **56.37** | **29.07** | **3.73** | **109.23** |
