# Supplementary material for: Fine-scale movement patterns and habitat selection of little owls (Athene noctua) from two declining populations
Source: PLoS One. 2021 Sep 27;16(9):e0256608. doi: 10.1371/journal.pone.0256608 (PMC8476024; doi:10.1371/journal.pone.0256608)
Supplement: Attachment — Submitted filename: Response to reviewers 2.docx [file pone.0256608.s007.docx]

Dear Dr. Corti,

Thank you again for giving us the opportunity to resubmit our manuscript. We also would like to thank both reviewers again for providing us with additional constructive feedback. We have incorporated their suggestions in the revised version. We respond to each comment in detail in our response to the reviewers.

We hope that these additional changes will facilitate the decision to publish our study in PLOS ONE.

Sincerely,

Martin Mayer

PONE-D-21-11960R1
Fine-scale movement patterns and habitat selection of little owls (Athene noctua) from two declining populations
PLOS ONE

Dear Dr. Mayer,

Thank you for submitting your manuscript to PLOS ONE. After careful consideration, we feel that it has merit but does not fully meet PLOS ONE’s publication criteria as it currently stands. Therefore, we invite you to submit a revised version of the manuscript that addresses the points raised during the review process.

Both reviewers provided useful comments, please provide arguments to their questions in some of their doubts, especially with reviewer #2.

Please submit your revised manuscript by Sep 16 2021 11:59PM. If you will need more time than this to complete your revisions, please reply to this message or contact the journal office at [plosone@plos.org](mailto:plosone@plos.org). When you're ready to submit your revision, log on to <https://www.editorialmanager.com/pone/> and select the 'Submissions Needing Revision' folder to locate your manuscript file.

Please include the following items when submitting your revised manuscript:

- A rebuttal letter that responds to each point raised by the academic editor and reviewer(s). You should upload this letter as a separate file labeled 'Response to Reviewers'.
- A marked-up copy of your manuscript that highlights changes made to the original version. You should upload this as a separate file labeled 'Revised Manuscript with Track Changes'.
- An unmarked version of your revised paper without tracked changes. You should upload this as a separate file labeled 'Manuscript'.

If you would like to make changes to your financial disclosure, please include your updated statement in your cover letter. Guidelines for resubmitting your figure files are available below the reviewer comments at the end of this letter.

We look forward to receiving your revised manuscript.

Kind regards,

Paulo Corti, Ph.D.
Academic Editor
PLOS ONE

Journal Requirements:

Please review your reference list to ensure that it is complete and correct. If you have cited papers that have been retracted, please include the rationale for doing so in the manuscript text, or remove these references and replace them with relevant current references. Any changes to the reference list should be mentioned in the rebuttal letter that accompanies your revised manuscript. If you need to cite a retracted article, indicate the article’s retracted status in the References list and also include a citation and full reference for the retraction notice.

** We have checked our reference list to ensure it is complete and have now detected any papers that have been retracted.

Reviewers' comments:


Review Comments to the Author

Please use the space provided to explain your answers to the questions above. You may also include additional comments for the author, including concerns about dual publication, research ethics, or publication ethics. (Please upload your review as an attachment if it exceeds 20,000 characters)

Reviewer #1: Thanks to the authors for addressing all of my comments on the first version of the manuscript.
Overall, i find your responses satisfactory and certainly understand your issue with small sample size affecting the addition of extra variables into your analyses such as weight of the birds.

** We would like to thank the reviewer for this positive feedback.

I do think however, that the paper could benefit from explaining more about food supplementation. I do still feel that although you have made it clearer throughout about the supplementation, there is a lack of discussion about it. You state the findings of the paper have implications for conservation of little owls, which they definitely do, but perhaps state that even with supplementation they are struggling - so that is not a solution. So for e.g. lines 454 - 456 add on something like ", even with the current regime of supplementation" - because that's true isn't it? even with supplementation they are flying further and STILL not succeeding. I think perhaps the supplementation can be regarded as just an element of the study design but i think there is room to explore a bit why it's not working. You refer to the Kasper Thorup paper from 2010, early on where you state birds are food supplemented, but you could use this more to develop some discussion around it. I do realise you have stated your MS is long already, but i think you can do this succinctly and i think it would add something.

** We agree with this comment, and now added some more considerations in the discussion regarding food supplementation. Lines 432-433: ‘Large foraging distances by male owls also suggest that food provisioning alone was not sufficient to sustain the chick rearing, although it was previously shown to increase fledgling success [28].’ And further (lines 471-474): ‘The large movement distances of male owls in Denmark together with previous findings of rapid population declines [28, 29, 72] suggests that prey availability is insufficient to sustain the current Danish little owl population, even with the current regime of food supplementation.’

Further to this, I really only have a few minor comments on wording, and have indicated these below.

lines 96-97: your wording in this additional section you have added in is not quite right, where you have written ..'these two populations that both consisted of...' - the populations themselves do not consist of intensively cultivated farmland, the areas in which they live do. So i would write something like "...these two populations, both of which are situated/ inhabit areas of intensively cultivated farmland, but which differ in exact composition of crop types and in supplementation of feeding....'

** Corrected as suggested.

lines 386 - 391: there is perhaps some literature that supports what you're saying here - see Mitchell et al. 2019 Plos One.

** Yes, we have now added this reference.

line 414: 'decreased' not 'decreasing'

** Corrected as suggested.

Reviewer #2: The authors have done a good job of addressing most of my comments. My remaining suggestions are relatively minor and are mostly clarifications of my previous comments that need a little more consideration.

** We would like to thank the reviewer for this positive feedback and additional suggestions for clarification.

Regarding my comments on “nightly home range”, I still feel uncomfortable with the use of the language of “home range” here. The concept of a home range requires that an animal shows ranging behavior, e.g. crosses the same area multiple times and that the home range contains all resource requirements for that animal (Fleming et al., 2015; Calabrese, Fleming, & Gurarie, 2016). It is ultimately up to the Editor to make the decision here, but I think “nightly space use” would be more accurate and potentially less confounding to readers. Similarly, generally where distance travelled is reported in the literature as a straight-line distances between locations, it is referred to as “displacement” (Tucker et al., 2018; Noonan et al., 2019). These are changes suggested simply for clarity and consistency across the literature.

** We agree and have now adjusted the wording (space use instead of home range size and displacement instead of distance moved) throughout the manuscript.

Regarding my comments on Fig. 5. To re-phrase more clearly, how was relative probability of use calculated? Please add to methods and briefly state here in the caption.

** The relative probability of use was calculated based on estimates obtained from logistic resource selection functions. We have now added this in the methods and give a reference that gives a detailed background (lines 262-264): ‘We fitted logistic resource selection functions, using generalized linear mixed-effects models with a binomial distribution and a logit link, using the R package ‘lme4’ [50]’. And in the figure legend: ‘The relative probability of use (as estimated by logistic resource selection functions) by Czech (grey) and Danish (black) little owls for the different land cover types.’

Regarding my previous comments and the authors’ response about generalities in results (pasted below):
“Lines 344-376. Please make sure it’s clear whether you are talking about generalities in your results (same results across both study areas) or about results for one of the study areas. It may be useful to work this into your structure, e.g. speaking about generalities first and differences second (or vice versa).
** We acknowledge that it was party unclear whether we were talking about a specific area or a general patterns, and now clarified these parts (please see within the manuscript).”
Please have another try at this. Firstly, it would be more convenient if you refer to whereabouts in the manuscript these changes have been made, as for other responses. From looking through the results, I can find only one sentence that has been changed:
“Habitat selection partly differed between females and males, and between the two study areas. Relative to available locations obtained from correlated random walks (Fig 6 and S1 Fig), all owls selected for built up areas and avoided cereal, and neither selected for nor avoided pastures and road verges.”
Unfortunately, this makes the distinction between study areas and sexes even less clear. If habitat selection differed between females and males as well as the two study areas, what does “neither” refer to?
Please address my above comments by first reading through and identifying the multiple locations across the results where this confusion occurs (unclear whether results apply to one study area or both, or one sex or both), address them clearly and indicate in the response where in the manuscript this has been done.

** We agree that this was still unclear and have now reworked the entire paragraph to clarify which owls we refer to in which sentence. Lines 373-389: ‘*Regarding habitat selection (comparing used positions to available positions obtained from correlated random walks; Fig 6 and S1 Fig), all owls (of both sexes and study areas) strongly selected for built up areas, avoided cereal, and showed no selection or avoidance for pastures and road verges. For the other land cover types, habitat selection partly differed between females and males, and between the two study areas. In both areas, rape was avoided by males, but selected by females. Maize and forest were selected by Danish males and avoided by Czech males, and other arable crops were neither selected nor avoided by males of both populations. Fallow, maize, and other arable crops were either avoided by females (in both populations) or not available; i.e., not present in proximity to the nest (Fig 5). Forest was neither selected for nor avoided by Danish females and not available to Czech females. Danish males selected for proximity to roads whereas Danish females avoided proximity to roads (this variable was not included in the analyses of Czech owls), and all owls, apart from Czech females, selected for proximity to field edges (Table 2). Finally, male owls in Denmark increasingly selected for maize with increasing distance from the nest at the expense of the other land cover categories, whereas there was no clear pattern for males in the Czech Republic (S2 Fig).*’

Lines 259-260. “We fitted generalized linear mixed-effects models with a logit link…” I assume with the binomial family, add this.

** Corrected as suggested.

Calabrese, J. M., Fleming, C. H., & Gurarie, E. (2016). Ctmm: an R Package for Analyzing Animal Relocation Data As a Continuous-Time Stochastic Process. Methods Ecol. Evol. 7, 1124–1132.
Fleming, C. H., Fagan, W. F., Mueller, T., Olson, K. A., Leimgruber, P., Calabrese, J. M., & Cooch, E. G. (2015). Rigorous home range estimation with movement data: A new autocorrelated kernel density estimator. Ecology 96, 1182–1188.
Noonan, M. J., Fleming, C. H., Akre, T. S., Drescher-Lehman, J., Gurarie, E., Harrison, A. L., Kays, R., & Calabrese, J. M. (2019). Scale-insensitive estimation of speed and distance traveled from animal tracking data. Mov. Ecol. 7, 1–15.
Tucker, M. A., Böhning-Gaese, K., Fagan, W. F., Fryxell, J. M., Van Moorter, B., Alberts, S. C., Ali, A. H., Allen, A. M., Attias, N., Avgar, T., Bartlam-Brooks, H., Bayarbaatar, B., Belant, J. L., Bertassoni, A., Beyer, D., Bidner, L., Van Beest, F. M., Blake, S., Blaum, N., Bracis, C., Brown, D., De Bruyn, P. J. N., Cagnacci, F., Calabrese, J. M., Camilo-Alves, C., Chamaillé-Jammes, S., Chiaradia, A., Davidson, S. C., Dennis, T., DeStefano, S., Diefenbach, D., Douglas-Hamilton, I., Fennessy, J., Fichtel, C., Fiedler, W., Fischer, C., Fischhoff, I., Fleming, C. H., Ford, A. T., Fritz, S. A., Gehr, B., Goheen, J. R., Gurarie, E., Hebblewhite, M., Heurich, M., Hewison, A. J. M., Hof, C., Hurme, E., Isbell, L. A., Janssen, R., Jeltsch, F., Kaczensky, P., Kane, A., Kappeler, P. M., Kauffman, M., Kays, R., Kimuyu, D., Koch, F., Kranstauber, B., LaPoint, S., Leimgruber, P., Linnell, J. D. C., López-López, P., Markham, A. C., Mattisson, J., Medici, E. P., Mellone, U., Merrill, E., De MirandaMourão, G., Morato, R. G., Morellet, N., Morrison, T. A., Díaz-Muñoz, S. L., Mysterud, A., Nandintsetseg, D., Nathan, R., Niamir, A., Odden, J., O’Hara, R. B., Oliveira-Santos, L. G. R., Olson, K. A., Patterson, B. D., De Paula, R. C., Pedrotti, L., Reineking, B., Rimmler, M., Rogers, T. L., Rolandsen, C. M., Rosenberry, C. S., Rubenstein, D. I., Safi, K., Saïd, S., Sapir, N., Sawyer, H., Schmidt, N. M., Selva, N., Sergiel, A., … Mueller, T. (2018). Moving in the Anthropocene: Global reductions in terrestrial mammalian movements. Science (80-. ). 359, 466–469.

While revising your submission, please upload your figure files to the Preflight Analysis and Conversion Engine (PACE) digital diagnostic tool, <https://pacev2.apexcovantage.com/>. PACE helps ensure that figures meet PLOS requirements. To use PACE, you must first register as a user. Registration is free. Then, login and navigate to the UPLOAD tab, where you will find detailed instructions on how to use the tool. If you encounter any issues or have any questions when using PACE, please email PLOS at [figures@plos.org](mailto:figures@plos.org). Please note that Supporting Information files do not need this step.

** We have uploaded our figures to PACE and submitted the final files.
