# Supplementary material for: Fine-scale movement patterns and habitat selection of little owls (Athene noctua) from two declining populations
Source: PLoS One. 2021 Sep 27;16(9):e0256608. doi: 10.1371/journal.pone.0256608 (PMC8476024; doi:10.1371/journal.pone.0256608)
Supplement: Attachment — Submitted filename: Response to reviewer comments.docx [file pone.0256608.s006.docx]

Dear Dr. Corti,

Thank you very much for giving us the opportunity to resubmit our manuscript. We would like to thank both reviewers for the time and effort they took to provide us with constructive feedback. We have incorporated their suggestions in the revised version. Below, we respond to each comment in detail.

We hope that these changes to the manuscript will facilitate the decision to publish our study in PLOS ONE.

Sincerely,

Martin Mayer

PONE-D-21-11960
Fine-scale movement patterns and habitat selection of little owls (Athene noctua) from two declining populations
PLOS ONE

Dear Dr. Mayer,

Thank you for submitting your manuscript to PLOS ONE. After careful consideration, we feel that it has merit but does not fully meet PLOS ONE’s publication criteria as it currently stands. Therefore, we invite you to submit a revised version of the manuscript that addresses the points raised during the review process.

Your manuscript got positive review from both referees. I found that all their comments are useful to improve your work, then pay attention to all suggestions they gave to you. Especially to comments suggesting a better explanation about your small sample sizes and reasons for choosing the populations and individuals.

Please submit your revised manuscript by Aug 05 2021 11:59PM. If you will need more time than this to complete your revisions, please reply to this message or contact the journal office at plosone@plos.org. When you're ready to submit your revision, log on to https://www.editorialmanager.com/pone/ and select the 'Submissions Needing Revision' folder to locate your manuscript file.

Please include the following items when submitting your revised manuscript:

- A rebuttal letter that responds to each point raised by the academic editor and reviewer(s). You should upload this letter as a separate file labeled 'Response to Reviewers'.
- A marked-up copy of your manuscript that highlights changes made to the original version. You should upload this as a separate file labeled 'Revised Manuscript with Track Changes'.
- An unmarked version of your revised paper without tracked changes. You should upload this as a separate file labeled 'Manuscript'.

If you would like to make changes to your financial disclosure, please include your updated statement in your cover letter. Guidelines for resubmitting your figure files are available below the reviewer comments at the end of this letter.

If applicable, we recommend that you deposit your laboratory protocols in protocols.io to enhance the reproducibility of your results. Protocols.io assigns your protocol its own identifier (DOI) so that it can be cited independently in the future. For instructions see: http://journals.plos.org/plosone/s/submission-guidelines#loc-laboratory-protocols. Additionally, PLOS ONE offers an option for publishing peer-reviewed Lab Protocol articles, which describe protocols hosted on protocols.io. Read more information on sharing protocols at https://plos.org/protocols?utm_medium=editorial-email&utm_source=authorletters&utm_campaign=protocols.

We look forward to receiving your revised manuscript.

Kind regards,

Paulo Corti, Ph.D.
Academic Editor
PLOS ONE

Journal Requirements:

Please review your reference list to ensure that it is complete and correct. If you have cited papers that have been retracted, please include the rationale for doing so in the manuscript text, or remove these references and replace them with relevant current references. Any changes to the reference list should be mentioned in the rebuttal letter that accompanies your revised manuscript. If you need to cite a retracted article, indicate the article’s retracted status in the References list and also include a citation and full reference for the retraction notice.

When submitting your revision, we need you to address these additional requirements.

1. Please ensure that your manuscript meets PLOS ONE's style requirements, including those for file naming. The PLOS ONE style templates can be found at

https://journals.plos.org/plosone/s/file?id=wjVg/PLOSOne_formatting_sample_main_body.pdf and

https://journals.plos.org/plosone/s/file?id=ba62/PLOSOne_formatting_sample_title_authors_affiliations.pdf

** We now updated the formatting of out manuscript accordingly.

2. We note that you have stated that you will provide repository information for your data at acceptance. Should your manuscript be accepted for publication, we will hold it until you provide the relevant accession numbers or DOIs necessary to access your data. If you wish to make changes to your Data Availability statement, please describe these changes in your cover letter and we will update your Data Availability statement to reflect the information you provide.

** We have now uploaded relevant data to Dryad. The DOI is: https://doi.org/10.5061/dryad.k3j9kd57m

3. We note that Figure 1 and Fig S1 in your submission contain map images which may be copyrighted. All PLOS content is published under the Creative Commons Attribution License (CC BY 4.0), which means that the manuscript, images, and Supporting Information files will be freely available online, and any third party is permitted to access, download, copy, distribute, and use these materials in any way, even commercially, with proper attribution. For these reasons, we cannot publish previously copyrighted maps or satellite images created using proprietary data, such as Google software (Google Maps, Street View, and Earth). For more information, see our copyright guidelines: http://journals.plos.org/plosone/s/licenses-and-copyright.

** The images in Fig 1 and Fig S1 are not copyrighted. The inset map in Fig 1 comes from an open access data source and the land cover data was collected by us directly. We now added this in the figure caption of Fig 1. Fig S1 only includes data that was collected (or simulated by us).

4. Please include captions for your Supporting Information files at the end of your manuscript, and update any in-text citations to match accordingly. Please see our Supporting Information guidelines for more information: http://journals.plos.org/plosone/s/supporting-information.

** Corrected as suggested.

Reviewers' comments:

Please use the space provided to explain your answers to the questions above. You may also include additional comments for the author, including concerns about dual publication, research ethics, or publication ethics. (Please upload your review as an attachment if it exceeds 20,000 characters)

Reviewer #1

Movement patterns of little owls - study discusses spatio temporal movement patterns of little owls using GPS in denmark and Czech Republic - supplemented and non-supplemented respectively.

Significant differences found between males & females in DK and males & m+f in CR, particularly in distance moved from nest, but reports more similar habitat selection between the two countries.
The authors used simulated correlated random walks to account for spatiotemporal autocorrelation.

Reviewer comments:

* Nice writing style, easy to read and well laid out.
* I would caution that although i completely understand that small sample sizes are the best that can be obtained sometimes, it's difficult to take a huge amount from 2 isolated populations. I think your reasons for choosing them could be better explained.

** We thank the reviewer for the positive feedback, and have now expanded our reasoning for choosing the two different populations. Lines 92-97: ‘*We compared nightly little owl home range size, spatio-temporal movement patterns, and habitat selection in surviving territories in Denmark with a local population in the Czech Republic that is also declining (Šálek and Schröpfer 2008), but not food-supplemented. We chose this comparison to contrast space use patterns among individuals of these two populations that both consisted of intensively cultivated farmland, but differed in the composition of crop types and regarding supplementary feeding, with the aim to understand space use to inform habitat restoration activities.*’

line 95-96 - would it also be useful to contrast with populations that are doing well?

** Yes, we very much agree with the reviewer and actually planned to also GPS-tag individuals in an increasing population in Schleswig-Holstein, Germany. However, we unfortunately did not obtain the required ethics permits, and thus could not add this comparison.

128 - weight of little owls - so 3.2g is <2.5% but perhaps it is useful to mention if danish owls weight less than other little owls across Europe.

** We have no evidence for this statement. Our data regarding body mass from Danish little owls are not different from owls of other populations.

136 - would it not be better to have a scale of rainfall? yes/no doesn't then differentiate between light drizzle and a downpour.

** We considered this option. The problem is that there was no night with strong rainfall and only very few nights with some rain, so we opted for the categorization. Including precipitation as numeric variable would not have changed our results (we initially checked this).

could you include weight of the bird in your LMMs? then you can actually start to link energetic cost to distance etc?
**We agree with the reviewer that including body mass could be interesting to explain the variation in movement patterns between individuals (especially within same-sexed individuals). However, considering that we only have two to four observations per group (i.e. 3 M and 3 F in CZ, and 4 M and 2 F in DK), we doubt that the variation in body mass would have allowed us to make biologically relevant inferences. Additionally, the small sample size did not allow us to include this many variables in our analysis.

286-288 - this result (interaction of area x sex) makes me think you should not have excluded the higher order
interaction from your other analysis (area x sex x time of night) - these interactions are interesting and perhaps there are differences due to chick brooding/ incubation?

** We agree with the reviewer that these differences are interesting, but argue that we took these things into account by analyzing the data separately for the two study areas (making it easier for the reader to interpret the results as compared to three-way interactions).

lines 318 - 323 - this section is a bit list-like. Could you frame it a bit differently? the figure (5) does a great job of showing what you mean, and i think it's a bit convoluted and hard to find out what's actually important. What i take away from it is that they select for different things, in the 2 different countries and the sexes also differ.

** We have now slightly modified this part to make it more clear, and as suggested, we added a sentence to state the main message, i.e. that habitat selection differed between sexes and areas (lines 368-372): ‘Habitat selection partly differed between females and males, and between the two study areas. *Relative to available locations obtained from correlated random walks (Fig. S1, 6), all owls selected for built up areas and avoided cereal, and neither selected for nor avoided pasture and road verges. Rape was avoided by males, but selected by females. Maize and forest were selected by Danish males and avoided by Czech males, and other arable crops were neither selected nor avoided by males.*’

line 330 -331 - this is a vague sentence, i think you could remove it. Start with the more interesting bit below that you have a clear indication that fixes of >15 mins apart would lose data. (as you also reiterate in 336-338).

** We somewhat disagree with the reviewers comment on this point, and argue that this sentence gives a general summary of the relevance of our findings.

354-356 - I think this is also why trying to see if bodyweight had any influence would be useful. I note in your table you also provide number of chicks. Did this have any influence on distance/time away from the nest?

** The number of chicks did not affect how far from the nest owls moved. We now tested this by including the interaction of sex and body mass in the model investigating the distance from the nest, but this effect was uninformative (both the interaction and the main effect of body mass only). We would like to leave this variable out of the analyses to not overfit our models, considering the small sample size (which would not allow for a meaningful biological interpretation of the general effect of body mass).

line 361 - "..densities close to the nest."
** Corrected as suggested.

373-374 - The fact that birds in DK are food supplemented does not actually feature very prominently throughout the methods and results section of the paper.
Perhaps it cannot, but by this point I had almost forgotten this is what you had done! You don't mention it when you talk about distance from the nest, etc.
What more can you say about its influence? So they still flew much further even though they were supplemented? I think there needs to be more information about this throughout.
** We now clarify in the methods that Czech owls were not food-supplemented (lines 118-119): ‘Czech little owls were not food-supplemented.’ And further in the results (lines 289-291): ‘*In total, we obtained 14,639 GPS positions of 6 individuals in Denmark (food-supplemented; 4 males and 2 females) and 6 owls in the Czech Republic (not food-supplemented; 3 males and 3 females), with GPS units obtaining data for 1-8 nights (Table 1).*’

378 - 380 - can you express structural diversity/crop height etc in one of your models using a metric of height differential or something like that? This is a great concluding sentence - It would be good if we were able to understand this from the results. This adds to literature about little owls so perhaps you can use a DSM/DTM/Lidar to create a metric and look at whether males/females in dk/cz select specifically for a strong height differential/short areas? Crop type as a factor does not always tell us this.
** This is a bit tricky, as we did not measure the exact vegetation height in all fields during the study. We doubt that such data would be obtainable via DTM or Lidar data (we are not aware of any such datasets at the relevant spatial scale) as the vegetation height is changing markedly from week to week during this time of the year. Thus, we are afraid that we have to restrict the discussion of vegetation height and structure based on vegetation type, although we generally agree with the reviewer that this would have been good to measure directly.

408 -409 - I don't think this is an impactful conclusion here. We KNOW that GPS can provide this information - this is well known, in fact you state it in the intro. Your impactful conclusions are those related to the habitat management and the stress that owls are under due to longer movements.
** With all due respect, we disagree with the reviewer on this point. The important message from this sentence is that fine-scale movement patterns obtained from GPS data can be used for conservation actions, which we elaborate in more detail in the preceding part of this paragraph. Thus, we would like to keep this sentence as is.

Potential other discussion comments: could you assess habitat available to populations in other areas? Do you know - since there are only 15 breeding pairs of little owls in DK, where they nest? And assess whether they are likely to behave in the same way?
** Considering that the habitat composition is similar in the other Danish areas where the remaining little owls nest, it is conceivable that they have similar space use patterns. However, without data to substantiate this speculation, we think that it makes little sense to include this in the discussion (also considering that the manuscript is quite long already; >8500 words).

Comments on figure legends - sometimes you put Czech birds first, sometimes Danish. I think you need consistency throughout, so either a is always Cz or Dk and b is always the other.

** We now changed the order in Fig. 2, so graphs for the Czech Republic consistently appear first in all figures.

Reviewer #2

I enjoyed reading this manuscript that leverages very high resolution (one position per minute) GPS tracking data over a short period (~1 week) to better understand fine-scale habitat selection and nightly movement behavior of little owls in Denmark and the Czech Republic, two regions where populations are declining. They report on fine-scale habitat selection features that were consistent across the individuals studied in the two regions: areas with short vegetation and structural diversity that facilitate prey abundance and capture success. They then provide some conservation insights for this species, including suggesting that increasing habitat heterogeneity by providing both low grassland and high-quality edge (e.g. hedgerows) will improve foraging habitat for little owls. They also identify road verges as a potential ecological trap for this species.

Though the study has its limitations in terms of extrapolating findings to the broader populations due to limited sample size and sampling period (as acknowledged by the authors), this study provides important insight into the fine scale movements of a species that is declining in multiple regions across its range. The manuscript is well-written and interesting to read, the analysis appears to be soundly done (although see some of my comments for alternative approaches – perhaps for future publications). I have some suggestions below that I believe will improve the clarity of the manuscript for the broad readership of PLOS ONE.

** We would like to thank the reviewer for the positive and constructive feedback.

Approach, aims and continuity: I felt that the aims of the project were a little hard to follow. From the title I was expecting a conservation focused paper, but the abstract seemed to indicate a methods paper. I would say the conservation approach is best, since you are not comparing the correlated random walk approach to other methods. Also, when we reached the predictions in the last paragraph of the introduction, it is not immediately clear to the reader how these specific predictions relate to the aims of the project. Similarly, it would be fantastic if it was simple for the reader to follow from your aims, to your predictions, through methods and results without having to check back on how it all fits together.

** We now partly revised our introduction to better link the background to our study aims and predictions. Lines 67-69: ‘*Here, we conducted a case study on the little owl (*Athene noctua*) to investigate how high-frequency GPS data can be used to investigate spatio-temporal movement patterns and habitat selection to inform conservation practices.*’

And further (lines 92-97): ‘*We compared nightly little owl home range size, spatio-temporal movement patterns, and habitat selection in surviving territories in Denmark with a local population in the Czech Republic that is also declining (Šálek and Schröpfer 2008), but not food-supplemented. We chose this comparison to contrast space use patterns among individuals of these two populations that both consisted of intensively cultivated farmland, but differed in the composition of crop types and regarding supplementary feeding, with the aim to understand space use to inform habitat restoration activities.*’

And finally (lines 105-107): ‘*Regarding habitat selection, we predicted that owls generally select for areas that are associated with high prey densities and allow for successful foraging, i.e. comparatively short vegetation of pastures.*’

Methods: these comments are intended for the interest of the authors and not as a suggestion for re-analysis. I appreciated your use of correlated random walks in the habitat selection analysis and enjoyed reading about this method that I have not used before. However, autocorrelation in the movement data is not addressed in the other analyses in your study (e.g. distance travelled, home range etc.). In case you’re not familiar with the work, the continuous time movement model approach (Calabrese, Fleming, & Gurarie, 2016) is a method designed to account for the autocorrelation structures within movement data in order to help estimate these other movement metrics (Fleming et al., 2015; Noonan et al., 2019). In line with this, you may want to refer to your nightly KDE home ranges as “nightly occurrence” or “nightly space use” given these areas are dependent upon the sampling regime (Fleming et al., 2016) and they show a single night’s space use, rather than longer term ranging behavior. For example, they are likely to be much smaller when measured with a 1 min sampling interval compared to if they were measured with a one hour sampling interval. Like I mentioned above, I don’t see a need to re-analyze, but the authors may want to acknowledge the influence of autocorrelation with reference to their other metrics (where this is not accounted for in the analysis) in the Discussion and mention why it is unlikely to influence their interpretation of results.

** We appreciate the reviewer’s suggestions regarding the continuous time movement model approach, which no doubt will be a useful tool for future movement analyses. Regarding the KDE definition, we already refer to them as ‘nightly home range size’, specifying that these estimates only refer to single nights. We agree of course that the KDE estimate could change depending on the sampling rate, but as we use the same fix rate across individuals and areas, this should not be an issue. Further, it was previously shown that KDE calculation is robust, given that the sample size is large enough (Börger et al. 2006, Wauters et al. 2007).

Lines 39-41. Individual responses are not discussed in the paper.
Lines 39-41. What is “inferior foraging habitat”?

** We have now removed these parts of the sentence.

Line 98. Replace “excessively” (value judgement), could replace with “X-X times larger”, or “much larger”

** We now write ‘at least twice the size’ to be more specific.

Lines 94-104. Link these predictions to your aims, to understand space use in order to inform habitat restoration activities
** We now revised this part to better link our study aims to the predictions. Lines 92-97: ‘*We compared nightly little owl home range size, spatio-temporal movement patterns, and habitat selection in surviving territories in Denmark with a local population in the Czech Republic that is also declining (Šálek and Schröpfer 2008), but not food-supplemented. We chose this comparison to contrast space use patterns among individuals of these two populations that both consisted of intensively cultivated farmland, but differed in the composition of crop types and regarding supplementary feeding, with the aim to understand space use to inform habitat restoration activities.*’

Lines 124-129. Important to mention during exactly which part of the breeding season the owls were tracked (nest-building, incubation, fledging etc) since it was such a short period. We also need to know whether this was the same period in both regions.. It would be useful to comment more on this during the discussion too, e.g. consistency between life cycle periods sampled across regions and whether you’d expect to find something different during other parts of the year.

** We now added the exact period (lines 136-137): ‘This period coincided with the fledgling period in both areas.’ However, we still did not know the exact age of the chicks, which might influence the food provisioning effort by the parents. As suggested by the reviewer, we now added this in the discussion (lines 424-426): ‘It is possible that the fledglings were not exactly the same age in the two areas (and among territories), which might have also affected owl movements due to varying food requirements of younger versus older fledglings.’

Line 222. Use “as” instead of “than”

** Corrected as suggested.

Lines 289-291. This issue, the relationship between number of GPS fixes and displacement, could be addressed by taking the CTMM approach described above (Noonan et al., 2019).

** We agree with the reviewer, but argue that our approach of including the number of GPS fixes into the model also accounts for biases in the estimation of hourly distances etc.

Lines 344-376. Please make sure it’s clear whether you are talking about generalities in your results (same results across both study areas) or about results for one of the study areas. It may be useful to work this into your structure, e.g. speaking about generalities first and differences second (or vice versa).
** We acknowledge that it was party unclear whether we were talking about a specific area or a general patterns, and now clarified these parts (please see within the manuscript).

Lines 358-361. Is this a diet analysis of food supplemented or un-supplemented owls?

** Food-supplemented owls. We added this information.

Line 366. Add the range of number of chicks here

** We added this.

Line 387. Replace “providing” with “provided”.

** We think the ‘providing’ is the correct term in this case.

Fig. 5. Could you please explain more about where the relative probability of use measurement comes from and how the avoidance threshold (grey dotted line) works?

** We have now added this information: ‘Available positions were created using correlated random walks. Values above the grey dashed line indicate selection, whereas values below the line indicate avoidance (for each used position we created 10 random positions).’

Literature referred to:

Calabrese, J. M., Fleming, C. H., & Gurarie, E. (2016). Ctmm: an R Package for Analyzing Animal Relocation Data As a Continuous-Time Stochastic Process. Methods Ecol. Evol. 7, 1124–1132.
Fleming, C. H., Fagan, W. F., Mueller, T., Olson, K. A., Leimgruber, P., & Calabrese, J. M. (2016). Estimating where and how animals travel: an optimal framework for path reconstruction from autocorrelated tracking data. Ecology 97, 576–582.
Fleming, C. H., Fagan, W. F., Mueller, T., Olson, K. A., Leimgruber, P., Calabrese, J. M., & Cooch, E. G. (2015). Rigorous home range estimation with movement data: A new autocorrelated kernel density estimator. Ecology 96, 1182–1188.
Noonan, M. J., Fleming, C. H., Akre, T. S., Drescher-Lehman, J., Gurarie, E., Harrison, A. L., Kays, R., & Calabrese, J. M. (2019). Scale-insensitive estimation of speed and distance traveled from animal tracking data. Mov. Ecol. 7, 1–15.

3. Have the authors made all data underlying the findings in their manuscript fully available?

The [PLOS Data policy](http://www.plosone.org/static/policies.action#sharing) requires authors to make all data underlying the findings described in their manuscript fully available without restriction, with rare exception (please refer to the Data Availability Statement in the manuscript PDF file). The data should be provided as part of the manuscript or its supporting information, or deposited to a public repository. For example, in addition to summary statistics, the data points behind means, medians and variance measures should be available. If there are restrictions on publicly sharing data—e.g. participant privacy or use of data from a third party—those must be specified.

[NOTE: If reviewer comments were submitted as an attachment file, they will be attached to this email and accessible via the submission site. Please log into your account, locate the manuscript record, and check for the action link "View Attachments". If this link does not appear, there are no attachment files.]

While revising your submission, please upload your figure files to the Preflight Analysis and Conversion Engine (PACE) digital diagnostic tool, <https://pacev2.apexcovantage.com/>. PACE helps ensure that figures meet PLOS requirements. To use PACE, you must first register as a user. Registration is free. Then, login and navigate to the UPLOAD tab, where you will find detailed instructions on how to use the tool. If you encounter any issues or have any questions when using PACE, please email PLOS at [figures@plos.org](mailto:figures@plos.org). Please note that Supporting Information files do not need this step.

*In compliance with data protection regulations, you may request that we remove your personal registration details at any time.* [*(Remove my information/details)*](https://www.editorialmanager.com/pone/login.asp?a=r)*. Please contact the publication office if you have any questions.*

**References**

Börger, L., N. Franconi, G. De Michele, A. Gantz, F. Meschi, A. Manica, S. Lovari, and T. Coulson. 2006. Effects of sampling regime on the mean and variance of home range size estimates. Journal of Animal Ecology **75**:1393-1405.

Šálek, M., and L. Schröpfer. 2008. Recent decline of the Little Owl (*Athene noctua*) in the Czech Republic. Polish Journal of Ecology **56**:527-534.

Wauters, L. A., D. G. Preatoni, A. Molinari, and G. Tosi. 2007. Radio-tracking squirrels: performance of home range density and linkage estimators with small range and sample size. Ecological modelling **202**:333-344.
